# Immune Cytolytic Activity as an Indicator of Immune Checkpoint Inhibitors Treatment for Prostate Cancer

**DOI:** 10.3389/fbioe.2020.00930

**Published:** 2020-08-06

**Authors:** Ze Gao, Yiran Tao, Yiming Lai, Qiong Wang, Zean Li, Shirong Peng, Junxiu Chen, Wenli Cai, Kaiwen Li, Hai Huang

**Affiliations:** ^1^Department of Urology, Sun Yat-sen Memorial Hospital, Sun Yat-sen University, Guangzhou, China; ^2^Guangdong Provincial Key Laboratory of Malignant Tumor Epigenetics and Gene Regulation, Sun Yat-sen Memorial Hospital, Sun Yat-sen University, Guangzhou, China; ^3^Department of Biochemistry and Molecular Genetics, School of Medicine, University of Virginia, Charlottesville, VA, United States; ^4^Department of Radiology, Massachusetts General Hospital, Harvard Medical School, Boston, MA, United States; ^5^Guangzhou Twelfth People’s Hospital, Guangzhou, China

**Keywords:** prostate cancer, immune checkpoint inhibitors, immune cytolytic activity, TCGA, tumor mutation burden

## Abstract

Immune checkpoint inhibitors (ICIs) treatment is becoming a new hope for cancer treatment. However, most prostate cancer (PCa) patients do not benefit from it. In order to achieve the accuracy of ICIs treatment in PCa and reduce unnecessary costs for patients, we have analyzed the data from TCGA database to find a indicator that can assist the choice of treatment. By analyzing the data of PCa patients with TMB analysis and immune infiltration analysis, we found the expression of immune cells in different immune infiltration groups. Commonly used markers of ICIs, expressed on CD8^+^ T cell, were highly expressed in the high immune group. Then we used the forimmune cytolytic activity (CYT) to determine its relationship with the target of ICIs treatment. Through the analysis of CYT score and the ligands of immune checkpoints, we found that there was a significant correlation between them. With the increase of CYT score, the expression of CD80/86, PD-L1/L2, TNFSF14, and LGALS9 also increased gradually. Similarly, CD8^+^ T cells were significantly increased in the CYT high group compared with the CYT low group in PRAD. The present research provides novel insights into the immune microenvironment of PRAD and potential immunotherapies. The proposed CYT score is a clinically promising indicator that can serve as a marker to assist anti-PD-L1 or other ICIs treatment. At the same time, it also provides a basis for the selection of other immune checkpoint drugs.

## Introduction

Prostate cancer (PCa) is the second most frequent cancer and there were almost 1.3 million new cases in the male population worldwide in 2018 (Bray et al., 2018). It is the fifth leading cause of cancer death among men. Over the past decade, the surgery and medical androgen deprivation therapy have been the primary treatment (Hellerstedt and Pienta, 2002). Despite initial robust responses to hormonal therapies, the majority of patients eventually develop advanced disease progress to castration-resistant prostate cancer (CRPC) (Heidenreich et al., 2014). Several new drugs have been approved for the treatment of CRPC in recent years, including androgen receptor axis-oriented (ARAT) drugs such as abiraterone acetate (ABI) and enzalutamide (de Bono et al., 2011). However, the patients with androgen receptor variant 7 (AR-V7) mutations are not sensitive to these drugs. Although many molecular mechanisms have been reported which take part in the pathogenesis of PCa, there is little known about the development and progression of PCa (Karantanos et al., 2015).

An emerging approach is the use and development of immunotherapy that is inherent to the body’s anti-tumor immune response. Immune checkpoint inhibitors (ICIs) are monoclonal antibodies against immune checkpoint molecules that have shown significant benefits in treating patients with a variety of cancers, opening new frontiers in cancer treatment (Topalian et al., 2012; Choi and Lee, 2020). Elevated evidences indicated that blocked the cytotoxic T lymphocyte associated protein 4 (CTLA4), and programmed death 1 (PD-1) or its ligand PD-L1 had demonstrated unparalleled therapeutic efficacy in cancers such as non-small cell lung cancer, metastatic melanoma and bladder cancer (Snyder et al., 2014; Garon et al., 2015). However, not all patients can benefit from immunotherapy. ICIs have no significant effect on prognosis compared with other treatments in many patients, especially in PCa (Lesterhuis et al., 2017). The choice of immune checkpoint treatment is mainly based on two points, namely tumor mutation burden (TMB) and immune cell infiltration. The TMB is the total number of mutations in a tumor specimen and it describes the status of genomic mutations (Chalmers et al., 2017). The TMB is a potential biomarker of ICIs in many cancer types. The higher the TMB, the more neoantigens the tumor expressed, and the more easily the tumor cells were recognized by the immune system (Nandakumar and Mills, 2019). An important feature of PCa compared to many other cancers is its relatively low burden of somatic mutations and reduced neoantigen expression (Lawrence et al., 2013). On the one hand, high TMB is required, on the other hand, sufficient immune cells are required to infiltrate the tumor site. On the basis of clinical responses to ICIs, tumors are classified as cold and hot (Lim et al., 2018). PCa has a low baseline of immune cell infiltration and a poor response to checkpoint inhibitor monotherapy (Comiskey et al., 2018). Unfortunately, fewer than 5% of CPRCs are effective for ICIs (Comiskey et al., 2018). At the same time, the treatment of ICIs are expensive. Therefore, understanding the composition and function of immune cells in patients with PCa, and looking for potential tumor markers are essential to effectively control cancer progression and immune response.

## Materials and Methods

### Gene Expression Data Sets

The RNA-seq data of 540 prostate samples, including 51 normal tissues and 489 tumor tissues, were obtained from TCGA database. The transcriptome expression profile were downloaded from TCGA website^1^. The ESTIMATE algorithm to calculate immune and stromal scores for each sample (Yoshihara et al., 2013). We obtained fragments per thousand base million (FPKM) of prostate adenocarcinoma (PRAD) patients from the TCGA database, and converted the FPKM value to Transcript Per Million (TPM) value. The CYT was calculated as the geometric mean of the granzyme A (GZMA) and perforin (PRF1) expression in TPM, which are dramatically upregulated upon activated CD8^+^ T cell (Rooney et al., 2015).

### Tumor Mutational Burden (TMB) Estimates

The masked somatic mutation data of PCa were downloaded from TCGA database. A total of 484 patients have somatic mutation information. The R package “maftools” (Mayakonda et al., 2018) was used to calculate the total number of somatic non-synonymous point mutations within each sample.

### Immune Cellular Infiltration Estimates

The abundance of tumor-infiltrating immune cells in PRAD samples was assessed using the CIBERSORT algorithm, which is a gene-based deconvolution algorithm that infers 22 human immune cell types and uses the characteristics of 547 marker genes to quantify the relative scores for each cell type (Newman et al., 2015). LM22 is the annotated gene signature matrix defining 22 immune cell subtypes, which is downloaded from the CIBERSORT^2^. The 22 immune cells include M0-M2 macrophages, resting dendritic cells, activated dendritic cells, resting mast cells, activated mast cells, eosinophils, CD8^+^ T cells, CD4+ naïve T cells, CD4+ memory resting T cells, CD4+ memory activated T cells, follicular helper T cells, regulatory T cells (Tregs), T cells gamma delta fractions neutrophils, B cells memory, B cells naïve, plasma cells, resting NK cells, activated NK cells, and monocytes. To improve the accuracy of the deconvolution algorithm, only the results with CIBERSORT *p* value < 0.05 were filtered and selected for the further analysis. The data were generated by using the “CIBERSORT” package in R language.

### Single-Sample Gene Set Enrichment Analysis (ssGSEA)

To investigate the immune infiltration landscape of PCa, we used single sample gene set enrichment analysis (ssGSEA) to estimate the population specific immune infiltration, which define an enrichment score to represent the degree of absolute enrichment of a gene set in each sample within a given dataset. Normalized enrichment scores (NES) could be calculated for each immune category. The ssGSEA analysis were performed by the “GSVA” package in R. Based on the results of the ESTIMATE analysis, we performed an unsupervised cluster analysis on all patients and divided the patients into high, medium, and low immune score groups. Heatmap and clustering were generated by using the “pheatmap” package in R language.

### TIMER Database Analysis

TIMER is a tool that can analyze immune infiltration in different cancer types and can analysis with tumor-infiltrating immune cells (TIICs). TIICs included B-cells, CD4 + T-cells, CD8^+^ T cells, dendritic cells, macrophages and neutrophils^3^ (Li et al., 2017).

### Gene Set Enrichment Analysis (GSEA)

Gene Set Enrichment Analysis (GSEA) was used to identify associated signaling pathways between low CYT score and high CYT score in PRAD. The 9996 sets were downloaded from the molecular signatures database (MSigDB) C5 GO gene sets collection^4^. The main statistics to examine the GSEA results were NES and nominal *p* value. In our study, GSEA was run with the default parameters (i.e., permutation number = 1,000, permutation type = “genesets,” and recompute time set to 1,000 times).

### Statistical Analysis

The statistics were executed using the R software (Version 3.6.2)^5^. A *P*-value of less than 0.05 was set as statistically significant for all the analyses. Spearman correlation analysis was used to evaluate the correlation between continuous variables. Variables between groups were compared by Wilcox *t* test.

## Results

### Mutational Genomic Landscape in PRAD

**FIGURE 1:**
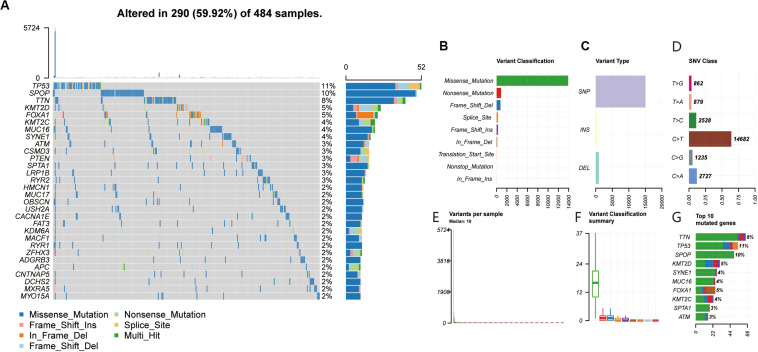
**(A,F)** Landscape of mutation profiles in PRAD samples. Mutation information of each gene in each sample was shown in the waterfall plot, in which various colors with annotations at the bottom represented the different mutation types. The figure showed the genes with the top 30 of tumor mutation burden. **(B,C)** The three variant types, and SNP showed more frequency than insertion or deletion. **(D)** The six variant types of single nucleotide variants, and C > T was the most common of SNV. **(E)** The number of altered bases in each sample. **(G)** The top 10 mutated genes in PRAD.

The waterfall map summarized high mutation genes and their mutation classifications in 484 PRAD patients. A total of 290 patients had the somatic mutation altered, accounting for 59.92% (Figure 1A). The missense mutation had the highest mutation frequency in the total mutation frequency (Figures 1B,F). The single nucleotide polymorphism (SNP) was more common variant type compared to DEL and INS (Figure 1C). Meanwhile, C > T had the highest incidence in the six variant types of single nucleotide variants (SNV), about 14682 times (Figure 1D). The median value of variants was 19 from 0 to 5724, much lower than other tumors (Figure 1E). In addition, the top 10 mutated genes were as follows: TTN, TP53, SPOP, KMT2D, SYNE1, MUC16, FOXA1, KMT2C, SPTA1, ATM (Figure 1G). From the gene cloud, genes with a mutation frequency of more than five were plotted (Supplementary Figure S1B). The font size is proportional to the number of mutations. To further research the relationship between high mutated genes, the co-occurrence and exclusive relationship were shown in Supplementary Figure S1A. The FAT3 was significantly correlated with KMT2C, *p* < 0.001. According to the above research, we found that the TMB in PCa was lower compared with other tumors. This means that it is more difficult for the infiltrated immune cells to recognize cancer cells.

### Immune Cells Infiltration in Tumor and Normal Tissues

**FIGURE 2:**
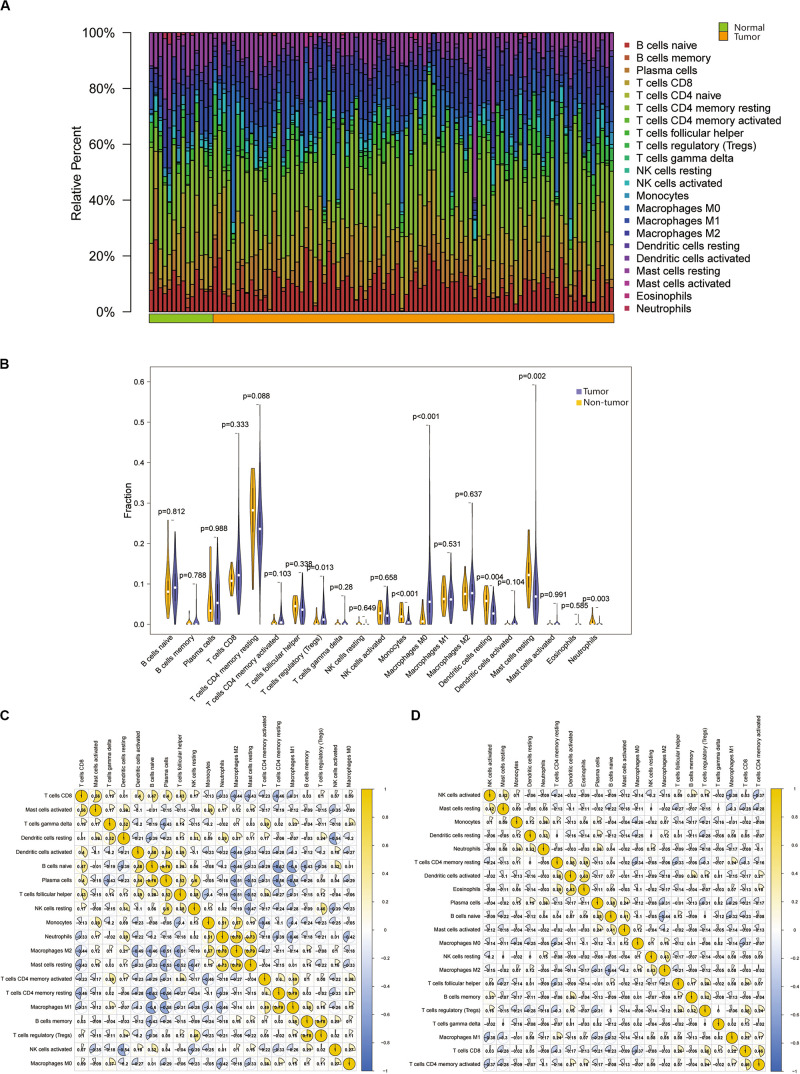
**(A)** Relative proportions of 22 TIICs subpopulation in normal and PRAD samples. Stacked bar charts of samples ordered by cluster assignment. **(B)** The Violin plot exhibits the difference between CIBERSOFT immune cell fractions between normal tissues and tumor tissues by Wilcox *t* text. **(C,D)** The correlation matrix of all 22 immune proportions in the TCGA PRAD cohort, including normal samples, and tumor samples.

To study the infiltration of immune cells in tumor tissues and non-tumor tissues, we used the CIBERSORT to process the data. Among the total samples, 88 tumor and 14 non-tumor samples were eligible with CIBERSORT *p* < 0.05. The CD4 naive T cells were excluded, as they were almost absent in both tumor and non-tumor tissues (Supplementary Table S1). As shown in Figures 2A,B, the fractions of immune cells varied significantly among non-tumor group and tumor group. The T regulatory cells (Tregs) and macrophage M0 significantly increased in tumor tissue (*p* < 0.05). Likewise, monocytes, dendritic cell resting, mast cell resting and neutrophils significantly decreased in tumor tissues compared with non-tumor tissues (*p* < 0.05). Although the CD8^+^ T cells were more abundant in tumor tissues than normal tissues, this was not significant. If we can pinpoint this subset of PCa patients, this subset of PCa patients could benefit from ICIs treatment.

Based on the above results, we found that the proportion of macrophage M0 and Tregs in the tumor was significantly higher than that in the normal tissue. Therefore, we select them for further analysis. And then we obtained the correlation between immune cells in non-tumor tissues and tumor tissues, respectively (Figures 2C,D). The higher positive correlation with macrophage M0 were T cells gamma delta, T cells CD4 memory activated and NK cells activated, while the higher negative correlation were plasma cells, neutrophils and mast cells resting in non-tumor tissues. There was no positive correlation between macrophage M0 and other cells in tumor tissues, only T cells CD4 memory resting and T cells CD8 had negative correlation with it. In non-tumor tissues, Tregs were positively correlated with NK cells resting and B cells memory, while only B cells naïve were negatively correlated with Tregs. In tumor tissues, Tregs were highly correlated with T cells CD8, B cells memory and T cells follicular helper, and negatively correlated with plasma cells, T cells CD4 memory resting and dendritic cells.

Thus, we speculated that the change in immune cell infiltration rate directly reflected the difference in immune function between normal tissues and tumor tissues. This may also explain why most patients with PCa are not sensitive to ICIs therapy. These results also suggested that reduced infiltrated immune cell may play an important role in the development of PCa.

### Immune Phenotype Landscape in PRAD

**FIGURE 3:**
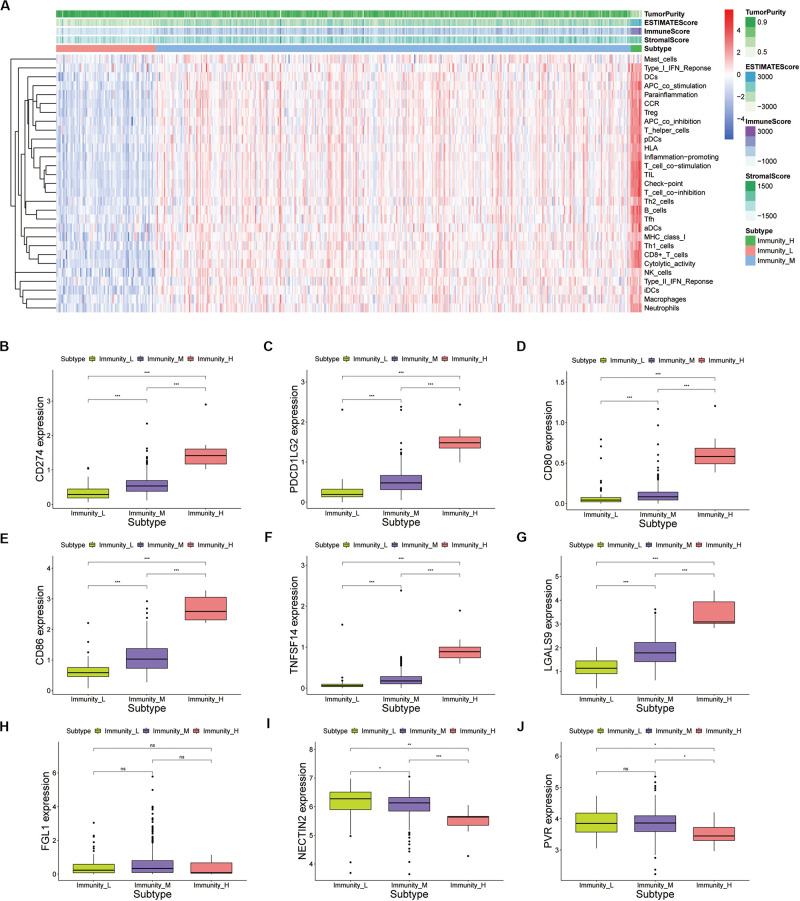
**(A)** Unsupervised clustering of PRAD patients using ssGSEA scores from immune cell types. The tumor purity, estimate score, immune score, and stromal score were shown as patient annotations in the top panel. **(B–J)** Inhibitory ligands of CD8^+^ T cells expressed by tumor cells were expressed in the high, medium and low immune groups of prostate cancer (*p* value, * < 0.05, ** < 0.01, *** < 0.001).

The degree of immune infiltration of each sample was assessed by ssGSEA. Unsupervised hierarchical clustering algorithm was performed based on scores of 24 immune cells in each sample. All samples were divided into three categories (Supplementary Figure S2A). These cluster samples were subjected to a heatmap of immune genes, and we divided them into high infiltration (*n* = 9), medium infiltration (*n* = 397), and low infiltration (*n* = 83) according to the infiltrated immune cells (Supplementary Figure S2B and Supplementary Table S2). In the PCa patients, the high immunity infiltration group only accounted for a small part, and the majority were the low- and moderate-immunity groups. The high infiltration group seemed to have more immune-active cells. The analysis of tumor microenvironment (TME) in PCa, including tumor purity, immune score, stromal score, and estimate score (Figure 3A). We found that high immune cells and stromal cells scores and reduced tumor purity in the high infiltration (Supplementary Figures S2D–G). Meanwhile, analysis of HLA expression in the three groups could also prove that the infiltration status of immune cells in different groups (Supplementary Figure S2C). Those results indicated that patients in the high immunity infiltration group could benefit more from ICIs treatment compared with the other two groups. Unfortunately, the high immunity infiltration group only accounts for a small part. Interestingly, the high and medium immune groups accounted for about 83.03%, indicating that ICIs treatment was still promising in PCa. Currently, numerous researches focuses on cytotoxic T cells because of their potent ability to kill tumor cells (Wahid et al., 2018; Lecis et al., 2019; Marra et al., 2019; Martinez et al., 2019). Meanwhile, targeting CTLA4 and PD1 has been successful in a variety of tumors (Schepisi et al., 2017). Lymphocyte activating 3 (LAG3) is the third inhibitory receptors to be targeted in the clinic (Andrews et al., 2017). Therefore, we explored the expression of inhibitory receptors in CD8^+^ T cells in prostate cancers including PD-1, LAG3, TIM-3, TIGIT, and BTLA. Meanwhile, competitive ligands for CTLA-4 receptors, including CD80, and CD86, were explored. The result showed that the CD80/86, PD-L1/L2, TNFSF14, and LGALS9 were significantly expressed in the high infiltration group (Figures 3B–G). The expression of FGL1, ligand of the LAG3, PVR and NECTIN2, ligands of TIGIT, were not significantly different in three groups (Figures 3H–J). We found that CD8^+^ T cell inhibitory receptors are significantly differently expressed in different immune groups (Supplementary Figures S3A–F). Although patients with PCa had relatively low mutation burden and relatively low immune infiltration, these markers were still significantly expressed in the high infiltration group. TIM-3, LAG3, TIGIT, and BTLA can be used as new immune checkpoints for drug development in PRAD beyond PD-1 and CTLA-4.

### Calculation and Verification of Immune Cytolytic Activity

**FIGURE 4:**
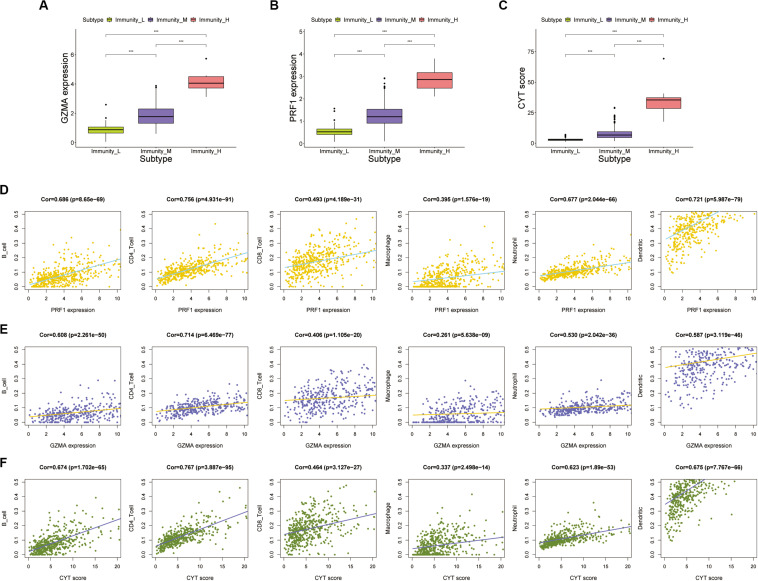
**(A–C)** The expression of GZMA, PRF1, and CYT in high, medium, and low immune groups (*p* value, *** < 0.001). **(D,E)** There was a significant correlation between the expression of PRF1 and GZMA and immune cells, including B cells, CD4 + T cells, CD8^+^ T cells, Macrophages, Neutrophils, and Dendritic cells. **(F)** There was a significant correlation between the expression of CYT score and immune cells, including B cells, CD4^+^ T cells, CD8^+^ T cells, Macrophages, Neutrophils, and Dendritic cells.

**FIGURE 5:**
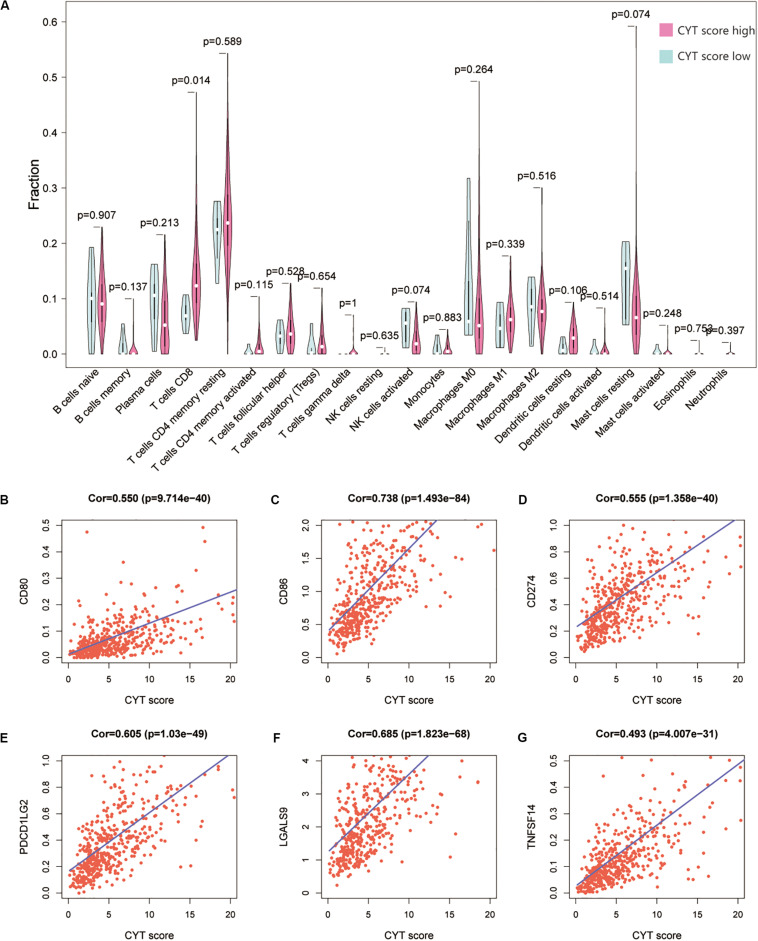
**(A)** The Violin plot exhibits the difference between CIBERSOFT immune cell fractions between low CYT group and high CYT group by Wilcox *t* text. **(B–G)** There was a significant correlation between the CYT score and CD8^+^ T cell ligand, including CD80, CD86, PD-L1, PD-L2, LGALS9, and TNFSF14.

**FIGURE 6:**
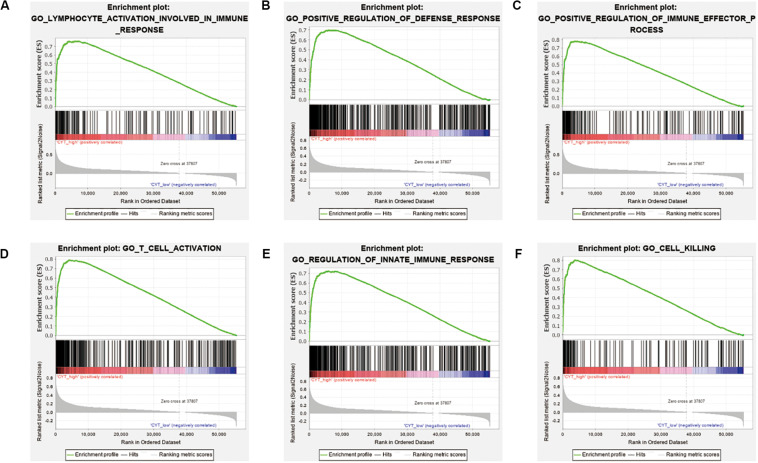
Different immune phenotypes between high- and low-CYT score groups in TCGA-PRAD cohort. **(A–F)** Gene set enrichment analysis for comparing immune phenotype between high- and low-CYT score groups. Significant enrichment of CYT score related GO terms in high CYT score group.

We calculated the transcription levels of GZMA and PRF1 to evaluate immune lymphocyte immune cytolytic activity in PCa. CYT score was calculated as the geometric mean of GZMA and PRF1. Based on the previous immune groups, we found that GZMA and PRF1 were significantly expressed in the high immune group. The CYT scores were similar in the low and moderate immune groups, and significantly decreased compared with the high immune groups (Figures 4A–C). This suggested that there was a sufficient reserve of CD8^+^ T cells in the high immune group. And the treatment strategy we need is to activate CD8^+^ T cells or to avoid its being inhibited. The TIMER web tool was used to assess the relationship between GZMA, PRF1 and CYT score and immune cells. It was obvious that they had a significant correlation with the infiltration of immune cells (Figures 4D–F). Therefore, we believed that the CYT score could be used as a basis for the degree of immune cell infiltration. We divided PRAD patients into CYT low group (*n* = 244) and CYT high (*n* = 245) group according to CYT score (Supplementary Table S3). Then, the infiltrated immune cells were compared between the two groups. To ensure the accuracy of the data, the data with *p* less than 0.05 after CIBERSORT calculation were retained. The CD8^+^ T cell infiltration was significantly higher in high CYT group than in low CYT group (Figure 5A). In order to verify the effectiveness of CYT score, we analyzed the correlation between CD8^+^ T cell inhibitory ligands and receptors and CYT score in PRAD patients. The results showed that CYT score was significantly correlated with these ligands (*p* < 0.001) (Figures 5B–G) and receptors (Supplementary Figures S3G–L). The PD-L1/L2, TNFSF14, and LGALS9 can be effective targets of ICIs treatment PCa in the future. GSEA was then conducted between the two groups, and more immune-related biological processes were found significantly enriched in the high CYT group, confirming our previous conclusion (Figure 6). The genes related with high CYT scores were more associated with T cell activation, positive regulation immune effector process, lymphocyte activation involved in immune response and others. The high CYT score conferred an enhanced immune phenotype, and could be used as an indicator of ICIs treatment and provided a basis for the development of new immune checkpoint drugs.

## Discussion

In recent years, cancer immunotherapy has made significant progress in clinical practice and has become another effective method besides surgery, radiotherapy, chemotherapy, and targeted therapy. Due to insensitivity of PCa to ICIs, we wanted to identify indicator or marker to assist in the choice of ICIs treatment in our study.

First, we analyzed the TMB in PCa using the TCGA database. We found that the TMB in PCa was not high compared to other types of cancer. Because of the low frequency of mutations, it was difficult for immune cells to accurately identify and find tumor cells leading to the immune escape. This could explain why some PCa patients do not benefit from ICIs treatment.

Next, we analyzed the infiltration of immune cells in patients with PCa. The result indicated that the T cell regulatory (Tregs) and macrophage M0 significantly increased in tumor cell (*p* < 0.05). The immunosuppressive effects of Tregs and unactivated macrophage M0 mediated the immune escape. By calculating the immune cell infiltration in tumor tissues, we divided the tumors into high, medium, and low immune groups. The majority of patients with PRAD were in the medium immune group, followed by the low immune group, and a small number in the high immune group. The CD8^+^ T cells are the main immune killer cells. Therefore, we investigated the ligands of inhibitory receptors on CD8^+^ T cells. The expression levels of PD-L1/L2, CD80/86, LGALS9, and TNFSF14 in high immunity group were significantly increased compared with the other two groups. And the expression level of HLA related genes was also significantly increased in the high immune group. These all confirmed why only a small percentage of patients with PRAD were sensitive to ICIs treatment.

Finally, to search for a marker associated with the inhibitory ligand of CD8^+^ T cells. We calculated the CYT score, based on the geometric mean of GZMA and PRF1 in TPM, to study the relationship with immune groups, immune cell infiltration and inhibitory ligand expression. We found a significant increase in CYT scores in the highly immune group, and significantly correlated with the degree of immune cell infiltration. The higher the CYT score, the more inhibitory ligands tumor cells expressed, leading to immune evasion. The patients with high CYT scores were more effective against checkpoint inhibitors such as PD-L1 and others than patients with low CYT scores. We also found that TNFSF14 and LGALS9 could be effective targets for ICIs in PCa. These results also provided a basis for the development of new drugs. Therefore, CYT score can be used as an auxiliary drug selection criterion in clinical practice to improve the efficacy of drugs.

## Conclusion

In summary, our study provides insight into understanding the novel potential role of CYT in ICIs treatment and its prognostic value. This also provides a new idea for immunotherapy of PCa. At the same time, it also improved the precision of ICIs treatment of PCa.

## Data Availability Statement

All datasets presented in this study are included in the article/Supplementary Material.

## Author Contributions

HH and KL designed the study and analyzed the data. ZG and YT wrote the manuscript and performed the data analysis. WC and YL critically revised the draft for important intellectual content. QW and ZL participated in the picture drawing and processing. SP and JC performed the statistical analysis. All authors read and approved the final manuscript.

## Conflict of Interest

The authors declare that the research was conducted in the absence of any commercial or financial relationships that could be construed as a potential conflict of interest.

## Footnotes

1 https://tcga-data.nci.nih.gov/tcga/

2 http://cibersort.stanford.edu/

3 https://cistrome.shinyapps.io/timer/

4 http://software.broadinstitute.org/gsea/index.jsp

5 https://www.R-project.org

## Supplementary Material

FIGURE S1 **(A)** The co-occurrence and exclusive relationship of the high mutation genes. **(B)** Genecloud plot showed mutation information of genes in PRAD. The size of the font represents the frequency of the mutation.

FIGURE S2 **(A)** Unsupervised hierarchical clustering algorithm in PRAD patients. **(B)** The heatmap of the all immune cells in high, medium and low immune group. **(C)** The HLA expression in the high, medium and low immune group. **(D–G)** The expression of the tumor purity, estimate score, immune score and stromal score in three immune groups (*p* value, *** < 0.001).

FIGURE S3 **(A–F)** Inhibitory receptor of CD8^+^ T cells expressed by tumor cells were expressed in the high, medium and low immune groups of prostate cancer (*p* value, *** < 0.001). **(G–L)** There was a significant correlation between the CYT score and CD8^+^ T cell inhibitory receptor, including CTLA4, PD-1, TIM-3, BTLA, TIGIT, LAG3, and TNFSF14.
