# Supplementary figures and images for: Immune Cytolytic Activity as an Indicator of Immune Checkpoint Inhibitors Treatment for Prostate Cancer
Source: Front Bioeng Biotechnol. 2020 Aug 6;8:930. doi: 10.3389/fbioe.2020.00930 (PMC7423880; doi:10.3389/fbioe.2020.00930)

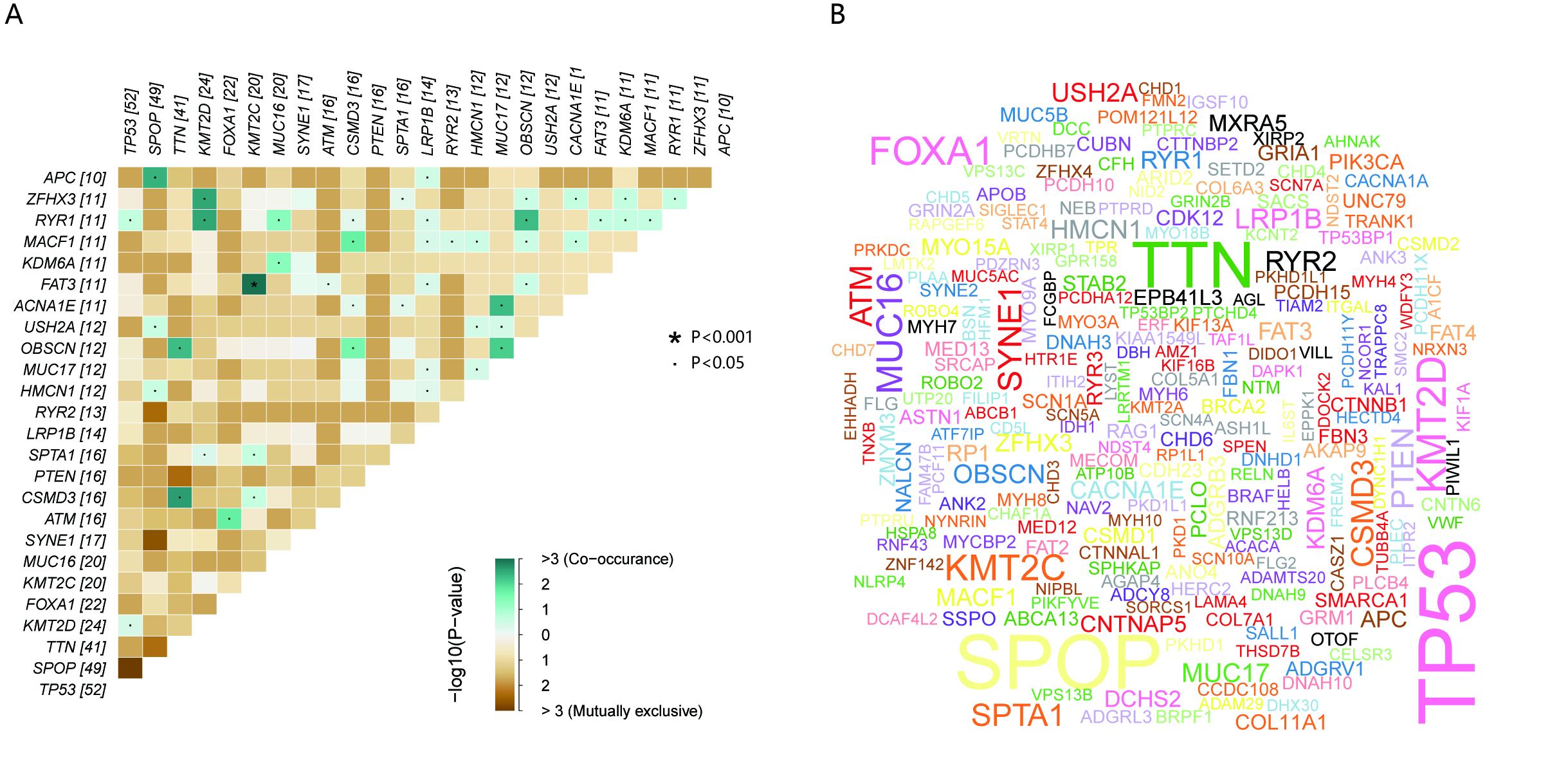

Supplement: FIGURE S1 — (A) The co-occurrence and exclusive relationship of the high mutation genes. (B) Genecloud plot showed mutation information of genes in PRAD. The size of the font represents the frequency of the mutation. [file Image_1.tif]

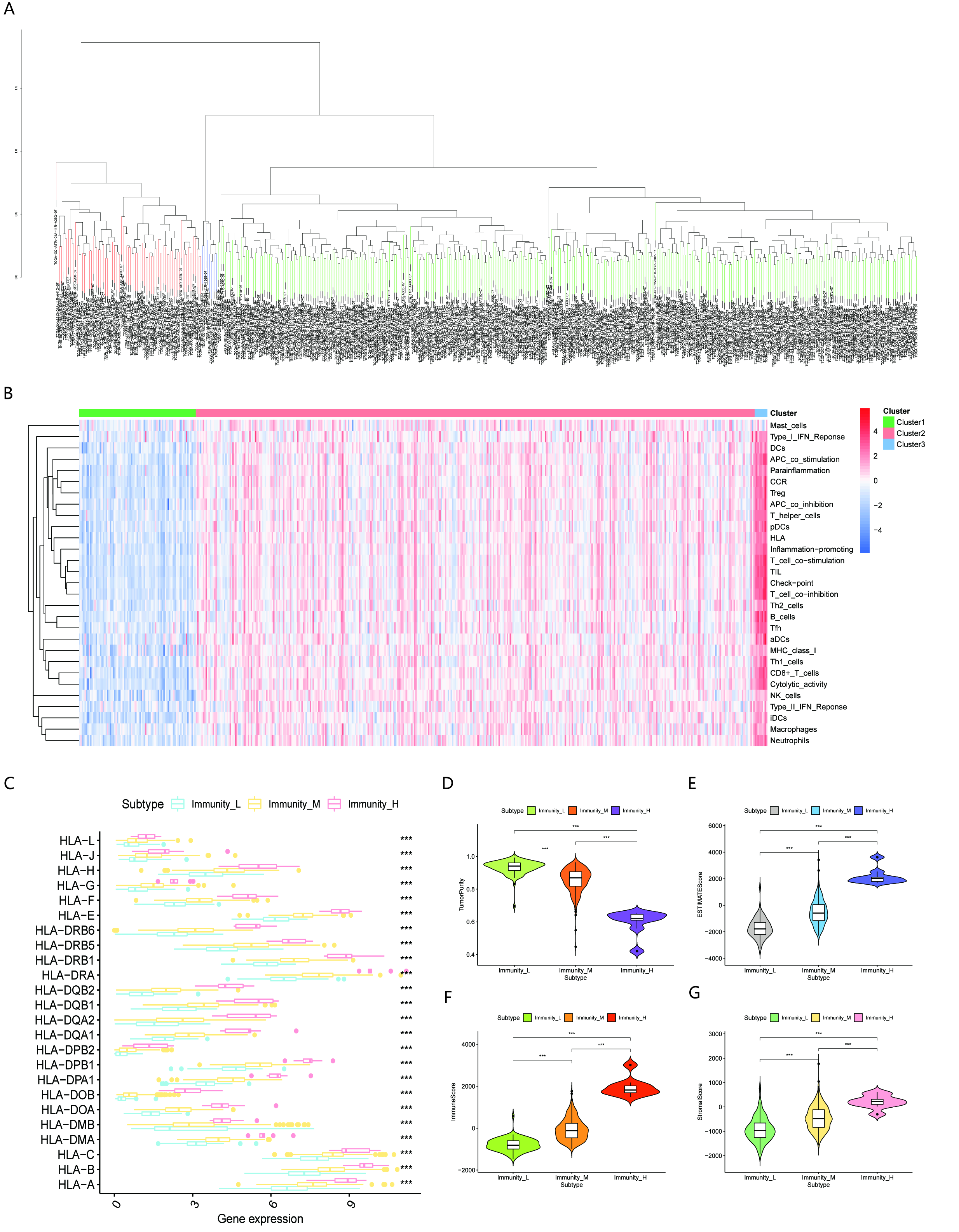

Supplement: FIGURE S2 — (A) Unsupervised hierarchical clustering algorithm in PRAD patients. (B) The heatmap of the all immune cells in high, medium and low immune group. (C) The HLA expression in the high, medium and low immune group. (D–G) The expression of the tumor purity, estimate score, immune score and stromal score in three immune groups (p value, *** < 0.001). [file Image_2.tif]

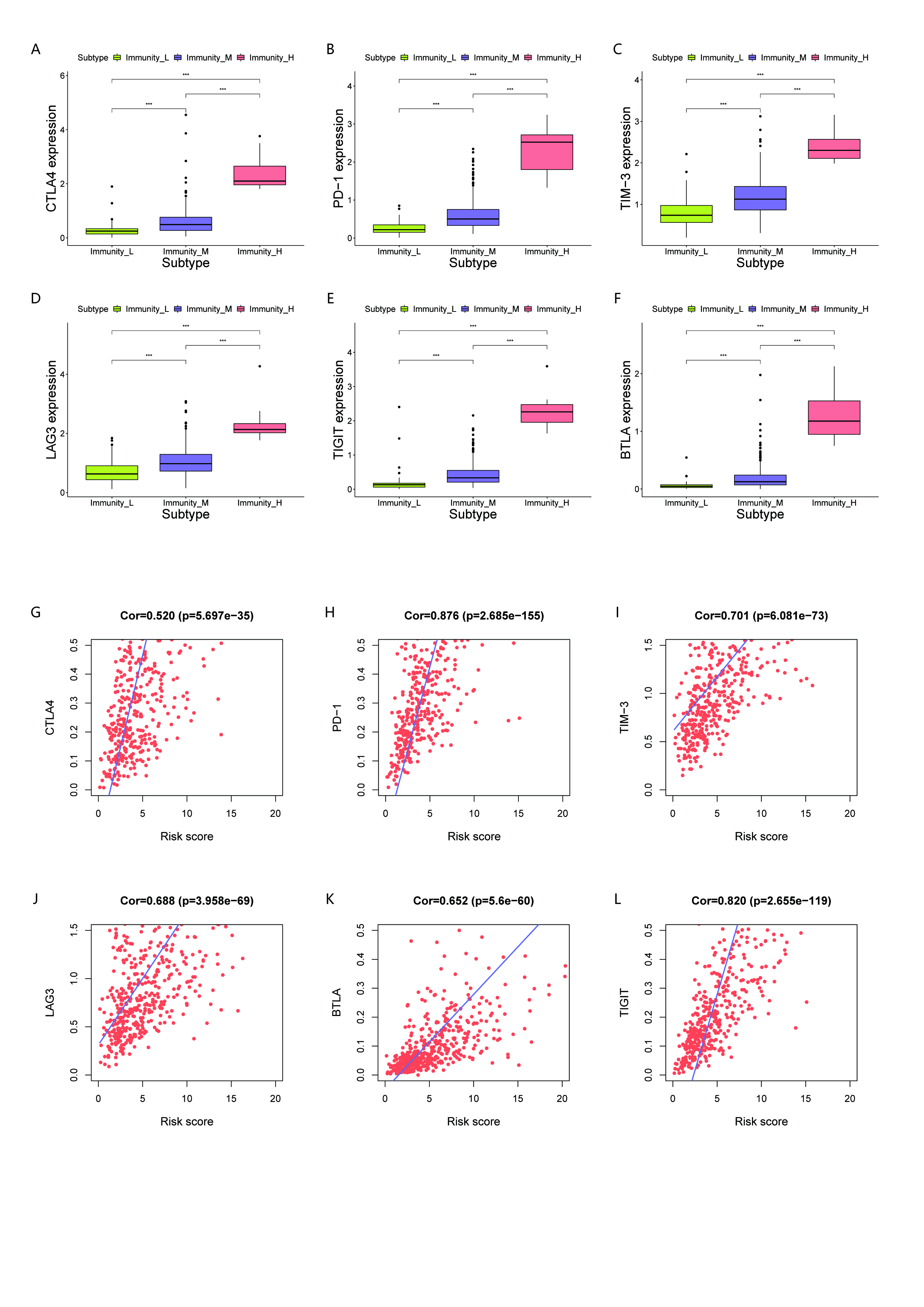

Supplement: FIGURE S3 — (A–F) Inhibitory receptor of CD8+ T cells expressed by tumor cells were expressed in the high, medium and low immune groups of prostate cancer (p value, *** < 0.001). (G–L) There was a significant correlation between the CYT score and CD8+ T cell inhibitory receptor, including CTLA4, PD-1, TIM-3, BTLA, TIGIT, LAG3, and TNFSF14. [file Image_3.tif]
